# Perceptions of the Sense of Security, Belonging, and Acculturative Stress among International Students in China during COVID-19 Outbreak—An Empirical Analysis

**DOI:** 10.3390/healthcare11081202

**Published:** 2023-04-21

**Authors:** Aijun Liu, Xiao Sheng, Innocensia Dickson Pato, Gladys Mutinda, Yanping Wu

**Affiliations:** 1 Jin Shanbao Institute for Agriculture and Rural Development, Nanjing Agricultural University, No. 1 Weigang, Nanjing 210095, China; liuaj@njau.edu.cn; 2 College of Economics and Management, Nanjing Agricultural University, No. 1 Weigang, Nanjing 210095, China; shengxiao623@sina.com (X.S.); innodpato@gmail.com (I.D.P.); 3 College of Public Administration, Nanjing Agricultural University, No. 1 Weigang, Nanjing 210095, China; gladysndungemutinda@outlook.com; 4 School of Overseas Education, Nanjing Xiaozhuang University, 41 Beiwei Road, Nanjing 210017, China

**Keywords:** international student, cross-cultural adaptation, COVID-19 pandemic, acculturative stress

## Abstract

Understanding the cross-cultural adaptation of students studying in foreign countries by exploring acculturative stress factors is crucial to ensure the smooth academic performance of the students and, in turn, to enhance the global reputation of their universities. Therefore, it is an area of interest for the authorities (Ministry) and the corresponding management of universities. Using a random sample of 138 international students in China, descriptive and logistic regressions were conducted to assess the levels and influence of acculturative stress factors on cross-cultural adaptation, specifically on international students’ sense of security and belonging during the COVID-19 pandemic outbreak. The results revealed that students were most concerned about homesickness, which obtained the highest mean score. The regression results indicated that the perception of fear and discrimination significantly impacted international students’ sense of security. The perception of fear, guilt, and how long the student stayed in China also significantly affected the sense of belonging. We argue that the reflections provided herein are essential for universities to improve how they manage and handle international students to mitigate the effects of acculturative stress, particularly when additional stressful conditions are present, such as the COVID-19 pandemic.

## 1. Introduction

The study of acculturation has gone through several stages of evolution. In particular, dimensions evolved from Berry’s two-dimensional model addressed the degrees of orientation on majority and cultural heritage [1,2,3] to emphasize acculturation strategies and adaptation while highlighting the importance of understanding their context. At present, the bidirectional model of acculturation put forward by [4] has been widely accepted in previous research. This study believes that the two dimensions of acculturation are the tendency of individuals to retain traditional culture and identity and seek communication with groups in other cultures. These two dimensions are independent of each other. An individual’s high identification with the main culture does not mean they abandon their original culture. The model theoretically explained and discussed the process of acculturation based on a definition of acculturation that is widely recognized. Therefore, acculturation refers to the gradual change in values, attitudes, and behaviors that occurs when individuals move to another culture and make continuous first-hand intercultural interactions [5,6]. Leaving behind one’s family, friends, and homeland and the acculturation process may be stressful and challenging [7], which often results in stress factors such as heightened psychosomatic symptoms, anxiety, identity confusion, marginalization, and depression. These stressors are collectively referred to as acculturative stress [8,9]. Acculturative stress is common among immigrants and sojourners. Previous studies have shown that these difficulties may contribute to reductions in an individual’s psychological [10,11] and physical health [12,13].

As one of the main groups of sojourners, international students attending higher education outside their native countries are more likely to struggle with acculturative stress due to the difficulties inherent in adjusting to their living conditions in a foreign country, which has attracted continuous scholarly attention. With limited exposure to the host country’s culture and lifestyle, university students are prone to experience higher levels of stress and depression [14,15,16]. The stress factors involved include language barriers, living away from families (limited social interaction), financial problems, and cultural shocks [17,18,19]. The literature on the impact of acculturative stress on international students’ well-being, for instance, shows that students who experience acculturative stress encounter considerable adjustment issues, such as a diminished sense of social belonging and pronounced levels of anxiety [20]. Belonging is an important aspect of acculturation and has been documented as a key component of academic and psychosocial success [3]. A more stable and lasting sense of belonging is much more crucial than a temporary feeling of belonging sprouting from thoughts, feelings, and behaviors [21,22]. According to [23], such students experience loneliness and/or isolation periods. If the acculturative stresses are not resolved for a long time, their academic life is bound to be affected. Furthermore, [24,25] indicates that when international students experience rejection or discrimination by the host culture, it becomes difficult to integrate into a new cultural environment, leading to deeper mistrust and further reducing their sense of security and belonging.

International higher education in China has been lucrative to many universities, with international education growing in size in our increasingly connected world. According to the statistics released by China’s Ministry of Education, 397,635 international students from 202 countries and regions had studied in 811 higher education institutions and scientific research in 31 provinces by 2019. These students created a complex and culturally diversified society by providing varied cultural values, mental outlooks, and social norms. Research on acculturative stress in international students in China is more representative and useful since the country welcomes students from both developed countries and developing countries [26]. Previous studies have shown that the release of acculturative stress was associated with individual perception and social support. The role of self-confidence in understanding acculturative stress and depression is crucial [27]. In terms of external support, the university needs to consider planning for services providing deep and long-term psychological help for international students [24] and to ensure that the appropriate interventions enhance international students’ performance during their stay in host countries. Research in this area needs to extensively explore the various contexts in which acculturative stress has an impact [14,15,16,28]. Apart from the drastic changes in lifestyle and the different aspects of academic and social life that international students are required to become accustomed to when living in a new country and culture, the outbreak of COVID-19 in late 2019 undoubtedly made it worse for international students who were in the cultural adaptation stage in a foreign country [29].

The COVID-19 pandemic has brought questions about individuals’ psychosocial well-being into focus. Studies have documented increases in depression [30], anxiety [31], and loneliness [32] in different countries. These stressors may aggravate the psychological burden of international students and increase their likelihood of suffering from various mental diseases [20,31,33]. Furthermore, the research on post-traumatic stress disorder (PTSD) and its psychological outcomes has expanded. Evidence from Malaysia, Yemen, Indonesia, Nigeria, Sri Lanka, and other countries shows that PTSD caused by COVID-19 is significantly associated with depression, anxiety, and insomnia at the level of latent constructs and observed variables [34]. The 22-item IES-R is a reliable screening instrument for measuring PTSD related to the COVID-19 pandemic and can be utilized to provide timely support for psychological health [35]. In addition to mental health, online education has attracted widespread attention in some countries. The evidence of semi-structured interviews from Jordan based on Moore’s theory of transactional distance shows the challenges involved in transitioning from traditional on-campus learning to online education [36]. 

Similarly, results from undergraduate and postgraduate students from Malaysia show that instructor performance, course evaluation, student characteristics, and system quality are four factors that influence students’ satisfaction with e-learning [37]. The evidence shows that the pandemic might have placed extraneous burdens of intercultural difficulties upon these students. During the outbreak, the need of the hour was to contain it in the infected areas: the Chinese government first sealed off Wuhan (the epicenter of the disease) and neighboring cities with high infection rates, then the country in general. Therefore, most international students remained trapped in China and faced its epidemic policy restrictions surrounding border control, visa, or flight during the lockdown [17,38]. Faced with all kinds of shock and stress from the restrictions on travel and social interaction activities, as well as the threat to their health presented by the pandemic, international students in China were forced to abide by the epidemic prevention measures during their stay in a foreign country whilst proceeding with academic endeavors [39]. The specificities of the situation added pressure to the pre-existing acculturative stress level patterns present before the disease outbreak [40], prompting research interest in this area. Studies have shown that working from home was a positive adjustment when dealing with epidemic prevention and control [40]; however, international students experienced insecurity, anxiety, and hopelessness [41,42]. Compared to the domestic students who had already gone home because of the Chinese New Year holiday, international students may have suffered more from the COVID-19 outbreak’s negative social and psychological outcomes.

This will be the case until the COVID-19 outbreak slows down; however, there are no signs of it happening soon as more waves and cases of Virus Mutations Variants rise in different parts of China and the world (WHO, 2021). The unique context of international student groups in foreign countries during the pandemic is an interesting area of research to investigate how acculturative stress affects emotional perception. Therefore, this study explores acculturative stress perception and the relationship between acculturative stress, and the sense of security and belonging the international students in China feel. We hope to provide suggestions to institutions and universities to improve their management of international students.

## 2. Methodology

### 2.1. Survey Design

Although early studies on cross-cultural adaptation mainly focused on using qualitative methods to determine the influencing factors of acculturative stress [43,44,45], in recent years, many studies have used quantitative methods to analyze psychological adjustment and social belonging [46,47,48,49]. Therefore, this study applied quantitative analysis techniques to synthesize the specific items of acculturative stress and investigated its influence on the sense of security experienced by international students in China, particularly during the COVID-19 pandemic period.

This study utilized a web-based, semi-structured survey questionnaire to collect information regarding cross-cultural adaptation before the outbreak of COVID-19, changes since the outbreak and to measure acculturative stress. The respondents were informed that participation was voluntary and that all collected information would be kept confidential and used for research purposes only.

The survey began by qualifying the respondents by determining if the respondent had the time to complete the survey and if they were in China during the COVID-19 pandemic outbreak from January 2020 to March 2020. The questionnaire included four parts: (1) conventional questions regarding the adaptation of international students, including how long they had been in China, their level of proficiency in Chinese, their rationale for studying in China, and their satisfaction with their institution; (2) perceptions and adaptation during the COVID-19 epidemic period; (3) acculturative stress in the four dimensions of homesickness, fear, guilt, and discrimination during the pandemic period; and (4) demographic information including gender, age, marital status, course, scholarship type, and country of origin. Based on the logistic regression model, this study analyzed the relationship between the two binary variables and the other factors.

### 2.2. Participants and Procedure

The survey started with the international students of Nanjing Agricultural University and adopted the snowball sampling method to distribute the questionnaire widely through WeChat groups of international students and on other social network applications. International students completely filled a total of 150 questionnaires with a valid sample of 138 responses that passed the screening test, which met the sample size requirements of logistic regression. Excluding a very small number of samples from the three continents of America, Europe, and Oceania, this study focused on international students from Asia and Africa, with a final valid sample size of 133. The data preparation process included questionnaire checking, editing, coding, transcribing, cleaning the data, and selecting a data analysis strategy. The research used STATA 16.0 software for the analysis.

Table 1 represents the demographics of the questionnaire respondents. The table shows that male respondents accounted for 75.2% of respondents. The proportions of respondents aged 18–25, 26–34, and 35–44 years accounted for 26.3%, 61.7%, and 12.0%, respectively. Most respondents were unmarried and came to China for higher degrees, with more than half studying for master’s and doctoral degrees. Regarding the continents of origin, the respondents came from, students from Asia accounted for 64.7% of respondents, while students from Africa accounted for 35.3%.

### 2.3. Statistical Analysis

We used a binary logistic regression model to analyze two psychological feelings that international students may have experienced during the pandemic period in China. Empirically, the model is expressed as follows:

*p*: the probability of the occurrence of the dependent variable;

X_n_: independent variables;

$\beta_{0}$: intercept;

$\beta_{n}$: coefficients of the independent variables;

ε: error term.

Specifically, the examined logistic regression model could be defined as:

1. Security = F_1_ [Homesickness, Fear, Guilt, Discrimination, Gender, Age, Marriage]
2. Prefer back = F_2_ [Homesickness, Fear, Guilt, Discrimination, Howlong, Gender, Age, Marriage]

For the dependent variable, we first investigated whether the respondents thought they were safe in China and understood this as the sense of security, denoted as Y_1_, which equaled one if the respondents chose Yes as the answer and zero otherwise. In addition, we also asked the respondents whether they would prefer to be back at their home to investigate their sense of belonging, denoted as Y_2_, which equaled one if the respondents chose Yes as the answer and zero otherwise.

For the explanatory variables, we used acculturative stress levels. Each dimension of the acculturative stress was further extended to encompass several particular items—i.e., subscales—which were adjusted to produce expressions that could better correspond to the student’s current situation based on the Acculturative Stress Scale for International Students developed by Sandhu and Asrabadi [14]: homesickness (5 items; for example, I miss the people and country of my origins), fear (3 items; for example, I fear that I cannot get quick treatment if I had novel coronavirus pneumonia), Guilt (3 items; for example, I feel guilty that I can get better medical treatment), discrimination (4 items; for example, I am treated differently because of my skin color). Each item was answered on a 4-point scale, where higher scores predicted higher stress levels among students and lower scores represented less acculturative stress. A continuous variable named Howlong was also added to the second regression, with values from 1 to 5; the larger the value, the longer the respondent had been in China. Additionally, several demographic characteristics were included in the regression as the control variables, including gender, age, and marital status.

## 3. Results

### 3.1. International Students’ Acculturative Stress in China

In Table 2, the mean scores show that international students in China experience significant concerns about each item. Higher mean scores represent more apprehension regarding the specific item area. We found that students were most concerned about homesickness, as it obtained the highest mean score. Among the items of homesickness, international students were most concerned about the last one: I miss the people and country of my origins. It obtained a 3.16 mean score, the highest overall score. International students expressed loneliness due to leaving their family, friends, or relatives in their countries of origin as they came to China for further studies. Several studies also highlight that homesickness is one of the obstacles to cross-cultural adaptation [28]. Fear was also a significant stressor among international students, with a mean of 2.57. The international students were most concerned about the item: I have some financial problems. There are several sources of income for international students, including scholarships from the CSC (China Scholarship Council), their home government, part-time jobs, and help from families. However, the epidemic outbreak made it difficult for students to find jobs, cutting off a source of income from part-time jobs.

Additionally, with the epidemic situation overseas becoming more serious, families may not have been able to help. Compulsory blockade measures also made it more difficult for international students to earn income from part-time jobs. For multiple reasons, more attention was paid to the financial aspect of the fear item. Regarding guilt, the highest mean was obtained by the following item: I feel guilty about leaving my family and friends behind. Adjusting to the host culture is generally conceived as infidelity and a betrayal of their own culture. The need for international students to adjust to another culture seems insincere to their culture and people, which may cause a sense of guilt. Unexpectedly, international students were least concerned about discrimination during the COVID-19 pandemic. This is contrary to the results of many studies [24,50], indicating that China may be more open and inclusive and that international students have benefited from the complete implementation of policies on this matter.

### 3.2. Descriptive Statistics for Logit Model

Table 3 shows the descriptive statistics for the variables used in the logit models. The dependent variable Y_1_ had a mean of 0.89, indicating that approximately 89% of international students felt safe in China during the outbreak. Similarly, it could be seen from the mean of Y_2_ that only approximately 36% of international students would have preferred to go back to their home country during the pandemic. The two binary variables together constituted the dependent variable of this study. According to the simple comparison of the means of acculturative stress, perceived homesickness obtained the highest score, with a mean of 2.98, indicating that many international students suffered from homesickness. All four means of the acculturative stress factors were under 3, showing that stress levels were low. The mean value of Howlong was 3.36, indicating that most international students had been in China for at least 1 year.

### 3.3. Regression Results

#### 3.3.1. Regressing 1: Logit Model of Security in China

The regression results with each variable’s estimated coefficients and *p*-values are displayed in Table 4 and Table 5. Table 4 shows that the coefficient of the perception of fear was positive and significant, while the perception of discrimination was statistically significant and negatively associated with the sense of security. The results showed that the other variables—homesickness, guilt, gender, age, and marriage—were not significant under the model specification.

#### 3.3.2. Regressing 2: Logit Model of Whether Would Prefer to Go Back to Home Country or Not

Table 5 displays the regression results regarding whether the students would have preferred to go back to their country of origin during the COVID-19 pandemic or not as the dependent variable. In acculturative stress, the perception of guilt was statistically significant and associated with the sense of belonging. The result indicated that higher scores on guilt led to a higher probability of international students being willing to return to their home countries, portraying a low sense of belonging. The guilt originated from the international students’ ability to obtain better medical treatment in China compared to those in their home country when they became sick/infected with the novel coronavirus. In addition, the coefficient of the variable How long was also negative and statistically significant, indicating that the longer the international students had been in China, the less likely they were to prefer going back to their home country. Several variables—homesickness, discrimination, gender, age, and marriage—seemed to have no significant correlation with the sense of belonging.

**Table 5:** Logit model for whether students would have preferred to return to their home country.

Variable Name	Coefficients	Standard Error
Homesickness	0.213	0.317
Fear	−0.496	0.335
Guilt	0.643 **	0.289
Discrimination	0.290	0.297
Howlong	−0.480 **	0.197
Gender	0.344	0.476
Age	0.094	0.354
Marriage	−0.096	0.458
Number of obs = 133		
Log Likelihood = −77.9456		
Wald chi2(10) = 17.47		
Prob > chi2 = 0.0256		
Pseudo R^2^ = 0.1038		

Note: ** is statistically significant at the 0.05 level.

## 4. Discussion

Drawing insights from the research on the acculturation of immigrants and sojourners, the main purpose of the present study was to investigate the acculturative stress levels of international students who were in China during the outbreak of the COVID-19 pandemic and the relationship between acculturative stress and their sense of security and belonging. The findings indicated that international students did experience the stress of cultural adaptability to some extent, and the mean of homesickness was the highest. Furthermore, we found that the perception of fear was positively related to the sense of security, while the coefficient of the perception of discrimination was negative and statistically significant. At the same time, the results of the other analyses on the sense of belonging were different: the length of time that international students had been in China was negatively associated with the sense of belonging, while the perception of guilt was positively associated with it.

This study found that international students often struggled with immigration and visa extension issues due to traveling restrictions [51]. The pandemic experience caused various forms of emotional challenges for international students who lacked adequate social support and, therefore, experienced loneliness, homesickness, anxiety, stress, and fear, especially during the lockdown periods [52,53]. However, the findings showed that these acculturative stress levels were not as high as expected. All the mean scores of the four dimensions were lower than 3, with 4 as the maximum. Several factors can explain these results. First, most international students have confidence in China’s stable social order and efficient policymaking in case of major emergencies such as the pandemic. This is because approximately 89% of the international students surveyed answered yes when asked whether they think they are safe in China. In the face of COVID-19, an emerging infectious disease, in addition to the classic non-pharmaceutical interventions such as isolation and quarantine, China also adopted strict mobility restrictions, including restrictions on travel between administrative districts [18]. Studies have shown that having formal social support can effectively alleviate psychological distress [19]. Moreover, other specific items of acculturative stress included information related to preventive procedures and regulation adherence. The lower mean scores also confirmed that students experienced a sufficient sense of security.

Many studies have focused on the relationship between acculturative stress and emotional stress. International students were reported to have a relatively high rate of depression compared to normal Chinese people [54], which was in line with the situation observed in other countries [55]. Afterward, research also found that China’s international students’ anxiety levels were higher than those of students in South Korea (49%) [56]. The present study investigated the correlation between acculturative stress and two perceptions of international students, i.e., their sense of security and belonging. The results showed that higher levels of perceived discrimination led to a lower sense of security. These results concord with the studies [24,25], indicating that when international students experience rejection or discrimination by the host culture, they struggle to integrate fully into this cultural environment, which might lead to deeper mistrust and a further reduction in their sense of security. As for the sense of belonging, the higher the level of guilt perceived, the more international students wanted to return to their countries of origin. The perception of guilt originated from the ability to obtain better treatment during the COVID-19 pandemic compared to relatives in the home country. In addition, the longer the international students were in China, the stronger their sense of belonging. This could be attributed to having more exposure to Chinese culture and services while living in China.

With the normalization of COVID-19, the post-epidemic era has put forward more empirical requirements for managing international students. It is necessary to strengthen the education of international students in China in daily health and safety measures and to improve their health awareness. Using our findings as a starting point, we should pay attention to the emotional state of international students in China and provide cross-cultural adaptation counseling services. If the acculturative stresses are not resolved for a long time, their study, work, and life will be affected. Therefore, a special counseling office can be set up to hire counselors with relevant professional knowledge and experience to provide “one-to-one” or “one-to-many” services for foreign students in China. We can also invite psychological experts to hold lectures on mental health in colleges and universities.

In addition, awareness of the social environment’s safety and stability helps attract international students to study in China, highlighting the importance of propaganda and ideological work. As the “home” of international students in China, colleges and universities should establish an effective working mechanism to convey accurate and useful information in time. At the same time, we should pay close attention to the negative information that international students easily misunderstand, take the initiative to understand the situation from their point of view and provide them with both sociological and psychological support services to calm their emotions and control their thoughts.

Moreover, strengthening these students’ language and cultural training can improve their environmental adaptability in China. Language adaptability is closely related to an individual’s sense of belonging and security in a foreign environment. Colleges and universities should improve international students’ ability to read, speak, and write Chinese in various ways to enhance communication skills by opening Chinese language teaching courses and organizing interesting activities related to Chinese culture.

Finally, to reduce the effects of acculturative stress on international students, there is a need to improve the support and counseling services available to enhance their sense of belonging and safety, especially during difficult situations such as the one brought about by the COVID-19 pandemic. In [26], the authors show the unsatisfying psychological situation of international students and the lack of research in China. Colleges and universities should carry out courses for international students to learn relevant regulations and rules for preventing and managing epidemics in the country. In addition, colleges and universities should also strengthen their career development services for international students, take the initiative to understand the academic and financial status of international students, and provide corresponding support guidance; moreover, they should open consulting services for international students to promptly capture their psychological status during national/global shocks, effectively dredge the pressures related to cross-cultural adaptation, and help international students have a happy life as students.

This study has some limitations. First, the sample size was relatively small and not representative of all the international students in China. Caution should be exercised in generalizing the findings to a larger population. Therefore, further research could broaden the scope of this study by recruiting large and representative samples. Secondly, the quantitative study relied on self-reported survey responses, which can be influenced by a social desirability bias. Thirdly, this study used only two perceptual, cognitive variables—binary variables—to investigate and measure the psychological conditions of international students, which might not be sufficient. Thus, future research should enrich the expression of relevant psychological conditions to make the results more accurate and focus more on exploring the impact mechanisms of acculturation on international students’ more diversified perceptions to permit causal inferences.

## 5. Conclusions

With the acceleration of the process of economic globalization and the increasing scale of international education, China, as an important host country in Asia, has attracted many international students from all around the world, which puts forward higher requirements for the shaping of a good learning environment and the management of international students. From the research results, the acculturative stress levels of international students in China were relatively low, showing a high average satisfaction. Meanwhile, the relationship between psychological feelings and acculturative stress gave university administrators and policymakers new ideas to improve their management levels. The results are also of value for researchers interested in exploring acculturation among international students in different contexts of higher education.

## Figures and Tables

**Table 1:** Demographic Characteristics of Respondents.

Variable	Classification Index	Numbers of Samples	Percentage (%)
Gender	Male	100	75.2
	Female	33	24.8
Age	18–25 years old	35	26.3
	26–34 years old	82	61.7
	35–44 years old	16	12.0
Marital Status	Married	47	35.3
	Not married	86	64.7
Continent	Asia	86	64.7
	Africa	47	35.3
Program	Undergraduate	11	8.3
	Associate degree	1	0.8
	Short Training	4	3.0
	Master’s degree	36	27.1
	Ph.D. degree	80	60.2
	Post-doctoral	1	0.8

**Table 2:** Items for acculturative stress with mean and standard deviation.

Items	Mean	Standard Deviation
**Homesi** **ckness**	2.98	0.82
I feel sad leaving my relative behind	2.96	0.97
Homesickness bothers me	2.93	1.06
I feel lonely	3.01	1.05
I feel sad living in unfamiliar surroundings	2.84	1.03
I miss my people and my home country	3.16	0.91
**Fear**	2.57	0.89
I fear for my safety because of my different race/skin color	2.55	1.09
I fear I cannot get quick treatment if I had novel coronavirus pneumonia.	2.56	1.10
I have some financial problems	2.60	1.12
**Guilt**	2.48	0.99
I feel guilty about leaving my family and friends behind	2.55	1.10
I feel guilty that I am living a different lifestyle here	2.52	1.11
I feel guilty that I can get better medical treatment	2.38	1.07
**Discrimination**	2.26	1.00
I feel that I receive unequal service treatment	2.38	1.12
I am treated differently because of my race/skin color	2.28	1.16
Classmates and/or friends are biased toward me	2.09	1.08
I feel that my community is discriminated against here in China	2.29	1.20
**Acculturative Stress**	2.57	0.77

**Table 3:** Descriptive statistics for variables.

Variable	Description	Mean	Standard Deviation	Min	Max
**Dependent Variables**					
Y_1_	Whether international students felt safe in China (Yes = 1, No = 0)	0.89	0.32	0	1
Y_2_	Whether international students would have preferred to be back home (Yes = 1, No = 0)	0.36	0.48	0	1
**Independent Variables**					
Homesickness	The mean scores of each item from 1 to 4	2.98	0.82	1	4
Fear	The mean scores of each item from 1 to 4	2.57	0.89	1	4
Guilt	The meanscores of each item from 1 to 4	2.48	0.99	1	4
Discrimination	The meanscores of each item from 1 to 4	2.26	1.00	1	4
Howlong	The amount of time that the respondents had been in China for	3.36	1.10	1	5
Gender	=1 if the respondents were Male, =0 if Female	0.75	0.43	0	1
Age	The age group of the respondents	1.86	0.60	1	3
Marital Status	=1 if the respondents were Married, =0 otherwise	0.35	0.48	0	1

**Table 4:** Logit model for the perception of being safe in China or not.

Variable Name	Coefficients	Standard Error
Homesickness	0.443	0.336
Fear	1.127 **	0.543
Guilt	−0.380	0.566
Discrimination	−1.388 ***	0.431
Gender	0.668	0.674
Age	−0.129	0.531
Marriage	0.100	0.684
Number of obs = 133		
Log Likelihood = −41.0308		
Wald chi2(9) = 22.02		
Prob > chi2 = 0.0025		
Pseudo R^2^ = 0.1243		

Note: **, and *** are statistically significant at the 0.05 and 0.01 levels, respectively.

## Data Availability

The datasets used and analyzed during this study are available from the corresponding author upon reasonable request.
